# Human Herpesvirus-6 Encephalitis After Hematopoietic Stem Cell Transplantation

**DOI:** 10.6004/jadpro.2014.5.5.8

**Published:** 2014-09-01

**Authors:** Heather L. Kasberg-Koniarczyk

**Affiliations:** Cleveland Clinic, Cleveland, Ohio

## CASE STUDY

Mrs. L. was in good health until she was diagnosed with acute myeloid leukemia (AML) at the age of 63. She had presented to her primary care provider with a productive cough and was found to have pancytopenia, with 16% myeloid blasts on a peripheral blood draw. She was admitted to a major metropolitan hospital for treatment of suspected AML. A bone marrow biopsy was performed, resulting in 44% myeloid blasts with complex cytogenetic studies [46,XX,der(16)t(1;16)(q21;q24)[2]/46,idem,t(4;16)(p12;q24)] and background ringed sideroblasts and dyserythropoiesis, implying underlying myelodysplasia in addition to AML. Molecular testing for FLT3, NPM1, and CEBPA was not performed, given the picture of antecedent myelodysplastic syndrome (MDS). The French-American-British classification system traditionally used for AML cases is no longer applicable for MDS in light of the system revisions in 2008.

Mrs. L. began standard 7+3 induction chemotherapy with daunorubicin and cytarabine (Table 1). Her bone marrow biopsy on day 14 was negative for residual AML, and her course was complicated only by tenosynovitis of the left foot, chemotherapy-induced nausea and vomiting, and culture-negative neutropenic fevers. Her posttreatment bone marrow biopsy was negative for AML but showed some residual dysplastic changes consistent with MDS; therefore, she received 5+2 consolidation chemotherapy with daunorubicin and cytarabine (Table 1). Following this regimen, Mrs. L. was rehospitalized for neutropenic fever. She developed sepsis from Streptococcus oralis, requiring intensive care for hypotension but no intubation. She completed a course of IV cefepime, recovered, and was discharged home.

**Table 1:**
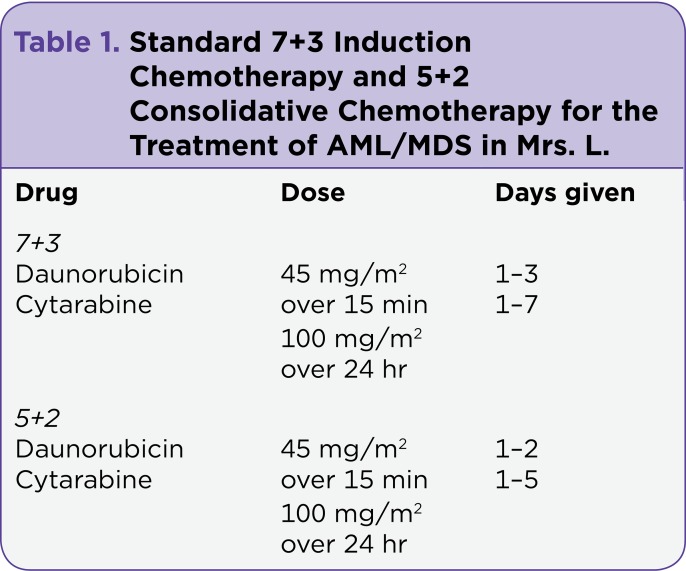
Standard 7+3 Induction Chemotherapy and 5+2 Consolidative Chemotherapy for the Treatment of AML/MDS in Mrs. L.

Due to the continued dysplasia in her bone marrow and the high risk for recurrence of AML, even after consolidative chemotherapy, Mrs. L. was referred for allogeneic hematopoietic stem cell transplant (HSCT), with the intent of a cure. Due to her age, she was found to be an acceptable candidate for a reduced-intensity chemotherapy preparative regimen using fludarabine, cyclophosphamide, antithymocyte globulin equine (ATG), high-dose methylprednisolone, and total-body irradiation (Table 2). The only source of donor stem cells that matched her human leukocyte antigen (HLA) was a multiple cord blood stem cell infusion consisting of two unrelated cord blood donations; cord A and cord B were infused without incident. Medications used to prevent graft-vs.-host disease (GVHD) were tacrolimus and mycophenolate mofetil.

**Table 2:**
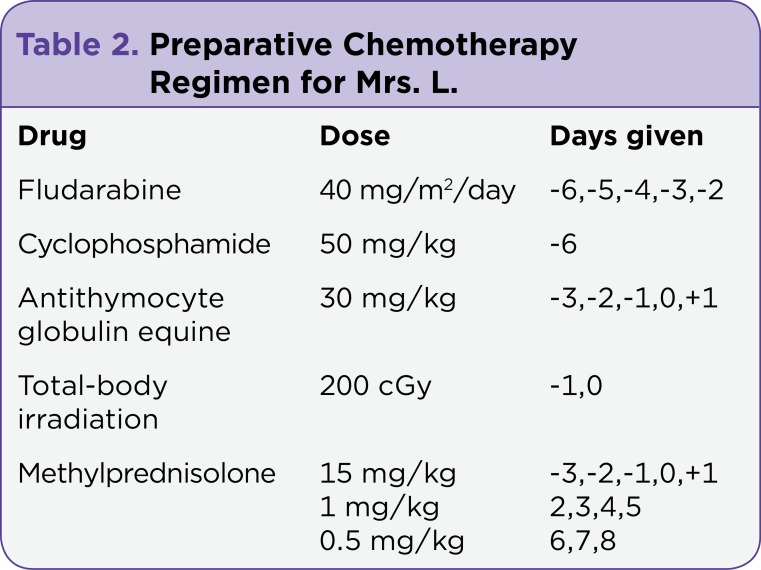
Preparative Chemotherapy Regimen for Mrs. L.

Mrs. L. became appropriately pancytopenic on day 5 after initiation of the HSCT preparatory regimen and was supported with blood and platelet transfusions. She developed oral mucositis on day 6 after the cellular infusion, as many HSCT patients do. She was treated with IV opioids and managed supportively with IV fluids and medications.

On day 3 posttransplant, Mrs. L.’s caregiver mentioned that she seemed confused. Upon examination, she was alert and oriented to person and place but not time. Her affect seemed unusual, and she responded to questions abnormally. At this time, she was neutropenic and severely immunosuppressed. Given the concern for sepsis, blood and urine cultures were drawn and a chest x-ray taken, but the results of all tests were negative.

The following day—day 4 posttransplant—tacrolimus was discontinued due to concern for possible posterior reversible encephalopathy syndrome (PRES). She underwent magnetic resonance imaging (MRI) of the brain (see Figure, part A), which revealed no abnormalities. On day 7 posttransplant, a lumbar puncture (LP) was performed, with all blood cell counts, chemistries, and infectious cultures negative.

**Figure 1:**
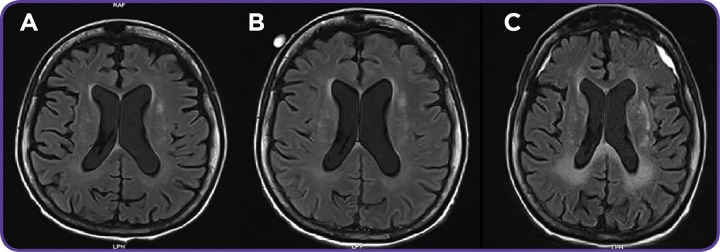
(A) Brain MRI on day 4 posttransplant with generalized atrophy and chronic, microvascular infarcts. (B) Brain MRI on day 11 posttransplant, stable exam compared with prior MRI. (C) Brain MRI on day 41 posttransplant with confluent white matter abnormalities representative of leukoencephalopathy, consistent with viral encephalitis. Also shown are bilateral frontal and posterior parietal subdural hematomas with little mass effect.

Mrs. L. began having neutropenic fevers along with progressive confusion the following day. She was oriented to person only. Neurology and infectious disease services were consulted. Blood and urine cultures were repeated, showing a vancomycin-resistant enterococcus (VRE) urinary tract infection (UTI). She was given daptomycin for 7 days, which resulted in clearance of the UTI but no improvement in her mental status. A second MRI of the brain was done on day 11 (see Figure, part B), which appeared to be normal.

Neurology recommended an electroencephalogram (EEG), which showed subclinical seizures. Mrs. L. was started on the antiepileptic drug levetiracetam, again with no improvement in her mental status. Additionally, she engrafted white blood cells, neutrophils, and other blood counts on days 25 through 28 posttransplant; a bone marrow engraftment study showed her blood was 100% cord A, signifying a successful engraftment, which can sometimes be difficult in cord blood transplant.

Mrs. L. eventually became nonverbal. A second LP was requested by neurology, infectious disease, and the transplant group, but her husband was hesitant for fear of causing her repeated discomfort. He eventually agreed, because she became progressively unresponsive. The LP was completed on day 27 posttransplant. Human herpesvirus-6 (HHV-6) was identified, with 5,300 viral copies/mL in the cerebrospinal fluid (CSF) and 41,000 viral copies/mL in the serum.

Mrs. L. was started on foscarnet therapy immediately. A repeat HHV-6 viral load was checked on day 14 following the initiation of foscarnet treatment. The CSF revealed no HHV-6, and there was a large reduction in the serum viral load, but there was no improvement in Mrs. L.’s neurologic status.

A third MRI of the brain was done on day 41 posttransplant, which showed new white matter changes consistent with leukoencephalopathy and bilateral frontal and parietal subdural hematomas with a mild mass effect (see Figure, part C); however, Mrs. L. was deemed a nonsurgical candidate by the neurosurgery service.

Neurosurgery, neurology, and infectious disease specialists agreed that inflammation from HHV-6 was likely the cause of her hematomas and that her prognosis seemed irreversible. After long discussions, her family chose palliative treatment alone. Mrs. L. passed away on day 48 posttransplant. Her death was likely caused by HHV-6 encephalitis with multiple subdural hematomas.

## Article

The International Committee on Taxonomy of Viruses (2011) describes HHV-6 as a member of the *Roseolovirus* genus, which is part of the herpesvirus subfamily. There are two distinct HHV-6 subspecies: HHV-6A and HHV-6B (Wang, Dong, Zhang, & Lu, 2008); however, most primary and reactivation complications arise from HHV-6B.

Within the first 3 years of life, most children have had a primary HHV-6B infection. This initial infection occurs through the salivary route in childhood, and the virus persists latently throughout life within the central nervous system (Zerr et al, 2014; Clark et al., 2006). Although the primary infection is usually asymptomatic, it can present as a mild and self-limiting fever (Kamble et al., 2007).

Reactivation of the virus in HSCT patients most typically occurs between 2 and 6 weeks following stem cell infusion; it occurs in 30% to 70% of allogeneic HSCT patients and is often asymptomatic and nonpathogenic (Zerr et al., 2005). Although reactivation of the virus is common, only a small number of patients develop encephalitis.

HSCT patients with HHV-6 encephalitis have variable long-term outcomes, ranging from full recovery with no residual neurologic deficits to major permanent neurologic dysfunction and even death (Seeley et al., 2007). HHV-6–related encephalitis in the HSCT population could result in substantial mortality (Ogata et al., 2013). Unfortunately, outcomes are difficult to evaluate due to the complex comorbid conditions that can result from HSCT. If other infection or bleeding issues are present, it can be challenging to determine whether death is a direct result of HHV-6 encephalitis.

Little is known about the morbidity and mortality of HHV-6 in relation to immunocompromised hosts, such as patients who undergo HSCT. Lesions seen on autopsy of patients with HHV-6 encephalitis are usually infiltrative of grey and white matter of the brain with edema and inflammation. Evidence of tissue necrosis, demyelination, and astrocytosis is seen histologically (Seeley et al., 2007).

## Risk Factors

Most risk factors are associated with patients in immunocompromised states such as those with human immunodeficiency virus (HIV) and acquired immune deficiency syndrome (AIDS). Among the HSCT population, HHV-6 reactivation has also been indirectly associated with GVHD and cytomegalovirus (CMV) reactivation (Wang, Dong, Zhang, & Lu, 2006; Zerr et al., 2012). Additional risk factors influencing the development of HHV-6–related complications post allogeneic HSCT include having an unrelated stem cell donor, having an HLA mismatched donor, receiving umbilical cord blood donor stem cells, and receiving certain monoclonal antibodies or posttransplant glucocorticoid treatments (Hentrich et al., 2004; Zerr et al., 2011).

## Clinical Presentation

HHV-6 can be associated with encephalitis, as evidenced by acute confusion or delirium, which quickly progresses to anterograde then retrograde amnesia within several days (Yamane et al., 2007). Seizures are seen in 40% to 70% of patients upon EEG, most reflecting subclinical seizures. Abnormalities are typically seen overlying the temporal or frontotemporal region of the brain. Occasionally, syndrome of inappropriate antidiuretic hormone secretion (SIADH) can be seen prior to the onset of confusion (Seeley et al., 2007). According to Bhanushali and colleagues (2013), symptoms of HHV-6 encephalitis usually present within the first 21 days after HSCT.

## Diagnosis

A diagnosis of HHV-6 encephalitis in HSCT patients is arrived at through a number of laboratory tests. The gold standard of diagnosis is direct detection of the virus using polymerase chain reaction (PCR) testing of the CSF, as serology is often falsely negative in HSCT patients (Seeley et al., 2007). Use of PCR detects the nucleic acid in the virus and can measure response to treatment when a quantitative assay is drawn. Reverse transcription PCR is not widely used, but it can determine the rate at which the HHV-6 virus is replicating. It is important to know that not all patients with HHV-6 encephalitis have a positive PCR in serum or CSF at presentation (Seeley et al., 2007). In the community, performing a viral culture is normally considered the gold standard for diagnosis; however, this process is lengthy and not recommended for deteriorating patients(Bhanushali et al., 2013).

## HSCT-Specific Workup

Analysis of CSF and serum for HHV-6 PCR quantification should be performed on all HSCT recipients presenting with confusion or delirium. Evidence of HHV-6 DNA in the bloodstream is not considered to be specific for HHV-6 encephalitis, but the results can be helpful, as high serum viral loads are likely to be associated with encephalitis (Tremblay, 2014). Viral loads of HHV-6 can vary widely from patient to patient; medial values range from 15,000 to 30,000 copies/mL and have been noted to be as high as 1,000,000 copies/mL (Ogata et al., 2013).

Magnetic resonance imaging is always done as well to confirm the diagnosis and to rule out other differential diagnoses. Hyperintensities of the limbic system are commonly seen, but imaging may be normal for weeks with known circulating HHV-6 in the CSF (Bhanushali et al., 2013). As computed tomography (CT) of the brain is normal in most cases, it is not helpful in this diagnosis (Noguchi et al., 2006).

## Differential Diagnosis

When working up a diagnosis of delirium with encephalopathy in the setting of HSCT, it is important to remember that HHV-6 is the most common cause of viral encephalitis. Other important differential diagnoses include bacterial and parasitic infections and viral causes such as Epstein-Barr virus, herpes simplex virus, CMV, varicella zoster virus, and JC virus—a type of human polyomavirus. It is also imperative to consider noninfectious causes when diagnosing a patient with encephalopathy posttransplant. Medications that can cause altered mental status, such as opioids or benzodiazepines, should be considered, as well as calcineurin inhibitors, such as cyclosporine and tacrolimus, which can cause PRES (Schmidt-Hieber et al., 2011).

## Treatment

Prompt treatment of HHV-6 encephalitis is important. Therapies consist of antiviral medications as well as anticonvulsants if seizures are present. Currently, there is no US Food and Drug Administration (FDA)‒approved treatment for HHV-6 encephalitis, although multiple studies show that ganciclovir and foscarnet have activity against HHV-6 in vitro (Zerr, 2014).

Response rates to therapy have been difficult to describe, and as such, there are currently no data identifying a clear superior response to foscarnet over ganciclovir. Therefore, current recommendations include using either foscarnet or ganciclovir in HSCT patients with HHV-6 encephalitis.

Foscarnet is dosed at either 60 mg/kg IV every 8 hours or 90 mg/kg every 12 hours, and ganciclovir is recommended to be dosed at 5 mg/kg IV every 12 hours. Unfortunately, there is no form of resistance testing available to date.

Side effects of these antiviral medications can be significant. Ganciclovir is associated with myelosuppression, which is challenging to manage in HSCT patients early in the transplant process who are awaiting engraftment. Foscarnet has its own array of toxicities. It often causes severe electrolyte depletion, is nephrotoxic, and can lower the seizure threshold in patients already at high risk for seizure.

Therefore, it is recommended that advanced practitioners (APs) monitor magnesium, potassium, and calcium serum levels closely and replace as indicated per institutional standards. To preserve renal function, hydration is often infused prior to each dose of foscarnet (Tremblay, 2014). The use of foscarnet requires caution in certain populations, such as patients with heart failure and acute kidney injuries, as the fluctuation of fluid status and electrolytes may worsen these conditions and put these patients at risk for cardiac dysrhythmia.

Currently, no studies have demonstrated the appropriate length of treatment for HHV-6 encephalitis. In patients whose CSF has cleared of detectable HHV-6, at least 21 days of therapy is recommended (Hill et al., 2012). For others who clinically progress and/or have residual detectable viral copies in the CSF, up to 6 weeks of therapy is given. It would be appropriate to switch agents if there is a lack of reduction in the viral load. Changing therapies as a result of toxicity may also be warranted.

Monitoring of weekly serum HHV-6 viral load by PCR is the most commonly used form of surveillance. Testing of the CSF for HHV-6 is usually repeated only if treatment failure is suspected, such as in the setting of a lack in reduction of serum HHV-6 viral load; the rationale is that there may be a possibility of drug resistance, which can be seen in other viral illnesses such as CMV (Zerr, 2014). It appears that generally, the level of HHV-6 in the CSF lags behind that in the serum in response to either therapy (Zerr, Gupta, Huang, Carter, & Corey, 2002). Currently, there is a lack of data on prophylaxis against HHV-6 reactivation in HSCT patients.

## Conclusion

Diagnosing and treating HHV-6 encephalitis can be difficult, as symptoms of this disease are usually present with few or no correlating diagnostic data. Continual testing should be performed on patients who are encephalopathic without a clear etiology. More studies are needed to optimize the treatment strategies for HHV-6 encephalitis, such as identifying a first-line therapy, establishing methods to evaluate the response to treatment, and discerning the preferred length of treatment. Patients with suspected HHV-6 or any viral encephalitis should be treated via a multidisciplinary approach, including providers of hematology/oncology, infectious disease, and neurology; speech, physical, and occupational therapies are also vital for helping patients to maintain their level of functioning.

There is hope that with the development of better detection abilities and continued aggressive supportive care, this often-fatal complication of bone marrow transplant may one day become just another treatable condition. l
